# Instrumenting Parkrun: Usefulness and Validity of Inertial Sensors

**DOI:** 10.3390/s25010030

**Published:** 2024-12-24

**Authors:** Rachel Mason, Yunus Celik, Gill Barry, Alan Godfrey, Samuel Stuart

**Affiliations:** 1 Department of Sport, Exercise and Rehabilitation, Northumbria University, Newcastle upon Tyne NE1 8ST, UK; rachel2.mason@northumbria.ac.uk (R.M.); gill.barry@northumbria.ac.uk (G.B.); 2 Department of Computer and Information Sciences, Northumbria University, Newcastle upon Tyne NE1 8ST, UK; yunus2.celik@northumbria.ac.uk (Y.C.); alan.godfrey@northumbria.ac.uk (A.G.); 3 Department of Neurology, Oregon Health and Science University, Portland, OR 97239, USA

**Keywords:** wearable technology, IMU, accelerometer, gait, running

## Abstract

The analysis of running gait has conventionally taken place within an expensive and restricted laboratory space, with wearable technology offering a practical, cost-effective, and unobtrusive way to examine running gait in more natural environments. This pilot study presents a wearable inertial measurement unit (IMU) setup for the continuous analysis of running gait during an outdoor parkrun (i.e., 5 km). The study aimed to (1) provide analytical validation of running gait measures compared to time- and age-graded performance and (2) explore performance validation. Ten healthy adults (7 females, 3 males, mean age 37.2 ± 11.7 years) participated. The participants wore Axivity AX6 IMUs on the talus joint of each foot, recording tri-axial accelerometer and gyroscope data at 200 Hz. Temporal gait characteristics—gait cycle, ground contact time, swing time, and duty factor—were extracted using zero-crossing algorithms. The data were analyzed for correlations between the running performance, foot strike type, and fatigue-induced changes in temporal gait characteristics. Strong correlations were found between the performance time and both the gait cycle and ground contact time, with weak correlations for foot strike types. The analysis of asymmetry and fatigue highlighted modest changes in gait as fatigue increased, but no significant gender differences were found. This setup demonstrates potential for in-field gait analysis for running, providing insights for performance and injury prevention strategies.

## 1. Introduction

Running is one of the most popular sports and recreational activities globally, consistently ranking among the top five favored sporting activities, with participation rates ranging from 7.9% to 13.3% across the six regions designated by the World Health Organization (WHO) [1]. Moreover, running serves as a fundamental element in numerous sports. The application of quantitative running gait analysis, both as a strategy to mitigate injury risks and as a metric for assessing performance, has attracted significant interest in the academic literature [2,3,4,5]. This approach plays a critical role in enhancing athletic performance and supporting the diagnosis and monitoring of injuries or medical conditions in clinical settings [3,6].

Traditionally, running gait analysis has been conducted in laboratory environments using various technologies, including 2D video analysis [7,8], 3D motion capture systems [9], force plates [10], instrumented walkways or mats, and treadmills [11,12,13]. While these methods provide accurate measurements of gait characteristics, they pose challenges, including high costs, the need for skilled operators, and the logistical burden of laboratory visits for participants. Furthermore, the controlled nature of laboratory assessments often fails to replicate the complexities of real-world running, limiting their practical application in both sports and clinical contexts [3].

Wearable technology offers a cost-effective, unobtrusive and lightweight solution to address the constraints associated with conventional methods of assessing running gait [14]. The growing acceptance and adoption of wearable technologies among sports practitioners, athletes, patients, and clinicians are noteworthy [15], with approximately 75% of runners using wearable technology for training optimization and distance recording [16]. Wearable technology utilizing accelerometers, gyroscopes, and magnetometers, either individually or in combination as a magneto-inertial measurement unit (MIMU), enable the quantification of running gait characteristics and have emerged as a viable running tools due to their portability and affordability [17].

Establishing the analytical validity and reliability of wearable technologies is essential for ensuring the accuracy of algorithms that measure running gait characteristics, thereby supporting confident performance evaluations and clinical decision-making in sports medicine [18,19]. While wearable technologies (e.g., Axivity Ax6, ViMove2, and DANU) have been extensively validated in indoor, controlled environments [2,20,21,22], the applicability and robustness of such systems in ecological, outdoor contexts remain underexplored. Building on initial analytical validation in specific cohorts, wearable technology can be further evaluated for usability and effectiveness across diverse environments, including both laboratory-controlled and real-world settings. Such evaluations deepen our understanding of running gait, offering valuable insights for clinical applications—such as managing neurological, musculoskeletal, and cardio-pulmonary conditions [23] and sporting contexts, including performance optimization, fatigue monitoring, and injury prevention [24]. This evolution represents a critical step in bridging the precision of laboratory tools with the practicality required for ecological applications.

This pilot study presents an inertial measurement unit (IMU) setup, to perform a continuous analysis of running gait during a parkrun, i.e., 5 km outdoors, extending prior validations performed in controlled environments [20,21,22]. The aim of the study is to present an exploratory analysis of usefulness and validity to (1) provide insight into instrumented running gait compared to (traditional) concurrent stopwatch time- and age-graded performance in healthy adults, and subsequently, to (2) develop performance validation of running gait measures through group differentiation comparing measures across different genders (i.e., known groups validity). Specifically, the system includes dual tri-axial IMUs (Axivity AX6) mounted on the talus joints of each foot for the measurement of temporal gait characteristics, including the ground contact time, swing time, and gait cycle duration. The IMU setup was tested on ten healthy participants during a 5 km parkrun, capturing the real-world running gait. These findings form part of a broader effort to validate wearable technology for use in ecological settings, enabling cost-effective running gait assessment without reliance on high-cost laboratory equipment. Section 2 describes the experimental setup and data collection process. Section 3 presents the analytical and performance validation results. Section 4 discusses the implications of our findings, followed by the study’s limitations. Section 5 concludes the work and highlights areas for future research.

## 2. Materials and Methods

### 2.1. Participants

A total of 10 participants (7 females and 3 males; age: 37.2 ± 11.7 years; height: 168.2 ± 4.6 cm; weight: 68.9 ± 8.6 kg) took part in this study. The inclusion criteria for the study were as follows: (i) participants had to be older than 18 years; (ii) they were required to train at least twice per week; and (iii) they must not have suffered from any injuries that could impact their running abilities in the last 6 months. All participants were supplied with informed consent forms and gave verbal and written consent before participating in the study. Ethical approval was granted by the Northumbria University Research Ethics Committee (Reference: 33358).

### 2.2. Data Collection

Data collection took place over two Parkrun sessions at a popular 5 km running route within the Newcastle-upon-Tyne region, UK. The single-lap course consisted of a mixture of trail paths and concrete paths, with 174 feet elevation gain. Participants were instructed to run the 5 km route at a self-selected speed. All runners completed the run (5 km ≈ 3.1 miles), and their average run time was 23.4 ± 5.7 min.

***Running Shoes:*** Participants wore their own running shoes. Two participants used carbon-plated running shoes whereas the remaining eight participants wore non-plated running shoes.

***Age-graded performance:*** Age grade performance percentage was calculated for each participant. Age grade performance in running is a quantitative method employed to standardize and compare the running performances of individuals across diverse age groups and sexes. It involves the adjustment of raw race times based on age and sex through the utilization of empirically derived factors, thereby facilitating equitable comparisons [25,26,27,28].

***Equipment:*** All participants were fitted with two wearable IMUs (AX6, Axivity, UK, https://axivity.com/ (accessed on 8 July 2022)), tri-axial accelerometer, and tri-axial gyroscope, 23.0 × 32.5 × 8.9 mm, 11 g) on the talus joint of each foot with medical tape. The placement of the IMU on the talus is crucial for accurately applying the zero-crossing methodology under examination (as described below). Monitoring the talus’s orientation offers an ideal representation of foot rotation during the running gait cycle [29], enabling precise assessment of foot strike patterns, pronation, and ground contact time (GCT). IMUs were programmed in Axivity’s OmGUI software suite, and the device’s accelerometer and gyroscope recorded data at a frequency of 200 Hz, with an operating range of ±16 g and ±2000 degree/s, respectively. Details of the Axivity AX6 specifications can be found on the Axivity website (https://axivity.com/product/ax6, (accessed on 8 July 2022)). Synchronization was ensured by trained expert manual segmentation of data using timestamps from the IMUs and the stopwatch reference time. Following the data collection, the raw data from the sensors were transferred to a Microsoft Windows 10 OS and analyzed using a custom-developed program in MATLAB^®^ version: 9.13.0 (R2022b). One participant’s right foot data could not be retrieved due to a sensor malfunction. Therefore, these data were not included in asymmetry characteristics.

### 2.3. Data Analysis

#### 2.3.1. Instrumenting Running Gait

Parkrun periods were manually segmented by trained experts from the IMU data recorded for each participant. This was performed using the timestamp information (e.g., starting time is 9 AM and finishing time is recorded for each runner). The data were processed using a Butterworth low-pass filter (4th-order Butterworth, cut-off frequency 20 Hz) applied to the vertical acceleration plane as well as the vertical and horizontal rotational velocity, effectively reducing signal noise. A previously validated method was used to identify initial contact (IC) (i.e., heel strike) and final contact (FC) (i.e., toe-off) using foot acceleration [30].

Subsequently, gait cycle duration (GC), the period between two consecutive *IC_moments* of the same foot; the GCT, the period during which the foot under observation is in contact with the ground; and the swing time (SWT), the phase where the foot under observation is airborne, were calculated based on the *IC_moments* and *FC _moments* timestamps for both feet (Equations (1)–(3)) [31,32]. We also calculated the stride-by-stride duty factor [33], which was the percentage of the stride duration where the foot under observation was in contact with the ground (Equation (4)). In addition to mean values, measures of asymmetry were also calculated using [34]. Asymmetry between the two sides is expressed as the degree of difference between the mean temporal characteristics of the left and right sides, represented as a percentage (Equation (5)) [35].

(1) $$GC=IC\;\left(k+1\right)-IC\;\left(k\right)$$

(2) $$GCT=FC\;\left(k\right)-IC\;\left(k\right)$$

(3) $$SWT=IC\;\left(k+1\right)-FC\;\left(k\right)$$

(4) $$Duty\;factor=\frac{GCT}{GC}\%$$

(5) $$Asymmetry=\sqrt{\left((GCT_{R}-GCT_{L}\right)/GCT_{L}\times 100{)}^{2}\;\;}=100\%$$

#### 2.3.2. Foot Strike Type Detection

Three prevalent foot strike types, heel strike, midfoot strike, and forefoot strike, are distinguished by the initial part of the foot to make ground contact. The foot strikes were analyzed by measuring the angular velocity in the sagittal plane around the mediolateral axis to determine the foot’s angle in the moment of IC using a validated algorithm [36]. Initially, angular velocity signal was low-pass-filtered (zero-lag 4th-order Butterworth filter) at 20 Hz to remove high-frequency movement artifacts. Then, the sagittal plane angular velocity signal was investigated around the moment of IC. Previous research [22,30,36] on foot strike types revealed distinct patterns: heel strikers exhibit a high negative peak in the rate of turn at foot strike, indicating foot roll from heel to toe; midfoot strikers demonstrate a more medium rate of turn; and forefoot strikers show a low negative peak and high positive peak, suggesting toe-to-heel roll.

### 2.4. Statistical Analysis

Statistical analysis was performed using the IBM SPSS Statistics program Version 25 (Armonk, NY, USA), with an alpha level of significance of *p* < 0.05. A descriptive analysis of all the study variables was conducted to find the mean and standard deviation. After this, the Shapiro–Wilk test was performed to check if the variables corresponded to a normal distribution.

#### 2.4.1. Analytical Validation

***Face Validity:*** The percentage change in temporal characteristics was calculated between the first half and the second half of the parkrun, as well as between the first minute and the last minute, to establish the slowing of running gait with increased time (i.e., a possible measure of fatigue, and changes in gradient). Pearson’s correlation coefficients were calculated to evaluate the strength and direction of linear associations between elevation and each running gait characteristic. Linear regression analyses were performed to assess the extent to which elevation explained each running gait characteristic, with the proportion of explained variance (R^2^) and significance levels (*p*-values) reported. To explore differences across elevation bands (low, medium, and high), elevation bands were categorized using quantiles, and ANOVA tests were conducted to determine whether the mean values of running gait characteristics varied significantly across elevation bands.

***Concurrent Validity:*** To compare running gait characteristics to the reference of stopwatch performance times (PT) and age-graded performance time (Age-PT), Spearman’s correlation coefficients were used. Correlations were interpreted as very weak (0–0.19), weak (0.2–0.39), moderate (0.4–0.59), strong (0.6–0.79), and very strong (≥0.8) [37].

***Reliability:*** To establish the reliability of running gait characteristics within a session, Spearman’s correlations were used to compare the gait characteristics in the first and second half of the run, and the first and last minute of the run. Reliability measures establish the consistency of temporal gait characteristics over time, a critical prerequisite for validating measurement systems. Reliable characteristics ensure observed changes reflect true biological variability rather than measurement error, which is foundational to analytical validation [18,38].

#### 2.4.2. Performance Validation

***Known Groups Validity:*** To establish whether running gait characteristics can differentiate known groups (i.e., groups known to have different running gait patterns), a *t*-test for independent samples was used when comparing the means of two groups (male (M) vs. female (F)) for performance time (PT), age grade performance based on performance time (Age-PT), and temporal characteristics. Furthermore, Mann–Whitney U tests and Kruskal–Wallis tests were performed to compare running gait characteristics between the different foot strike groups (HS, MS, and FS), and levels of Age-PT (above 70% and below 70%). The threshold for Age-PT was chosen based on standardized age grade practices, ensuring consistency with previous research while enabling effective differentiation of performance groups. This cutoff reflects biomechanical efficiency commonly associated with higher performance levels, as evidenced by significant differences in ground contact time and duty factor between groups [27,39].

## 3. Results

### 3.1. Analytical Validation

**Figure 1:**
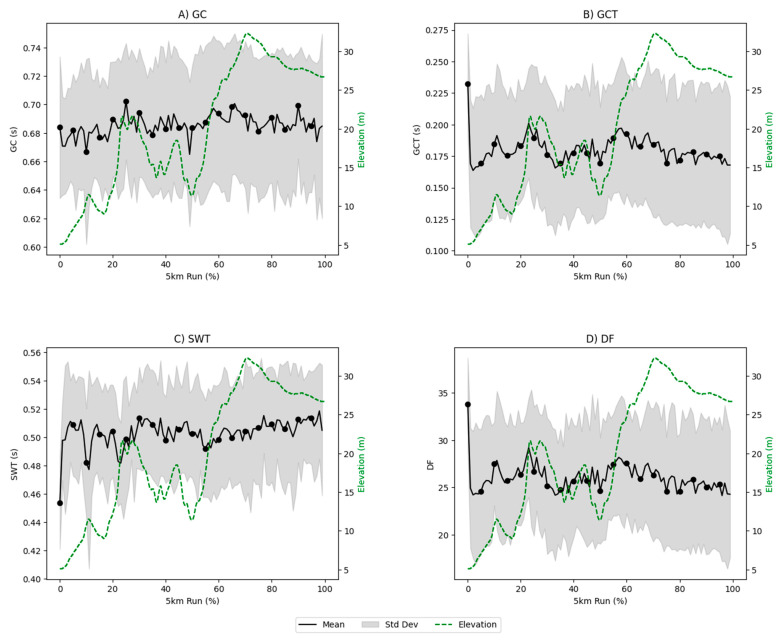
Time series analyses of running gait characteristics over the 5 km run (%). (**A**) Gait cycle, (**B**) ground contact time, (**C**) swing time, (**D**) duty factor. Each panel includes the mean (solid black line), standard deviation (shaded gray area), and elevation profile (dashed green line).

Table 1 provides an overview of the temporal characteristics (GC, GCT, SWT, and DF) and measured PT and Age-PT for each runner. Figure 1 depicts the time series analyses of the running gait characteristics, including GC, GCT, SWT, DF, and ASY-GCT, throughout the duration of the 5 km run (with elevation shown by the dashed green trace).

*Face Validity:* The analysis of the temporal characteristics (Figure 1) and their asymmetry (Figure 1 and Table 2) during the run reveal face validity. Throughout the run, there were observable increases in the mean values of the GC and GCT; specifically, GC increased by 1.01% from the first half to the second half and by 2.19% when comparing the first minute to the last minute. GCT showed a more substantial increase of 5.51% over the halves and 6.66% from the first to the last minute. SWT remained relatively stable with slight decreases, while the DF increased moderately, highlighting a shift in the distribution of the running cycle. The GCT and SWT asymmetries increased by 5.24% and 3.91%, respectively, from the first to the second half of the run, and there were even more pronounced changes from the first to the last minute. Finally, the DF asymmetry increased modestly by 0.75% in the second half and by 3.42% between the first and last minute (Table 2).

Figure 1 illustrates the temporal gait characteristics throughout the 5 km run, highlighting adaptations to elevation changes. For GCT, elevation explained less than 0.1% of the variation in GCT (R^2^ = 0.000, F(1, 998) = 0.19, *p* = 0.670). Additionally, differences in GCT across the elevation bands were not significant (F(2, 997) = 0.94, *p* = 0.391). GC showed a weak positive relationship with elevation. Elevation explained 0.6% of the variation in GC (R^2^ = 0.006, F(1, 998) = 6.37, *p* = 0.012), a statistically significant result at *p* < 0.05. Differences across the elevation bands were also significant (F(2, 997) = 3.18, *p* = 0.042), though the effect size appeared limited. For SWT, elevation explained 0.5% of the variation in SWT (R^2^ = 0.005, F(1, 998) = 4.62, *p* = 0.032), a statistically significant result at *p* < 0.05. However, the ANOVA results indicated no significant differences in SWT across elevation bands (F(2, 997) = 2.75, *p* = 0.064). In the case of DF, elevation explained less than 0.1% of the variation in DF (R^2^ = 0.000, F(1, 998) = 0.08, *p* = 0.774). The ANOVA results confirmed no significant differences in DF across elevation bands (F(2, 997) = 1.14, *p* = 0.319). ASY showed a weak positive relationship with elevation, which explained 0.6% of the variation in ASY (R^2^ = 0.006, F(1, 898) = 5.09, *p* = 0.024). These results were statistically significant at *p* < 0.05. However, the ANOVA analysis found no significant differences in ASY across elevation bands (F(2, 897) = 1.02, *p* = 0.361).

*Concurrent Validity:* The preliminary analyses demonstrate very strong positive correlations between PT and GCT (rho = 0.918, *p* < 0.05). Similarly, a strong (negative) correlation with Age-PT and GCT was found (rho = −0.964, *p* < 0.05). The mean SWT shows a weak correlation with PT and Age-PT (rho = −0.14, *p* = 0.101 and rho = 0.164, *p* = 0.145, respectively). Investigating asymmetry in relation to PT, Age-PT reveals that the correlations are weak and non-significant (*p* > 0.05), Table 2.

Regarding the foot strike patterns, no significant correlations were found with either PT or Age-PT, though heel strike showed a moderate, non-significant correlation with Age-PT (rho = 0.600, *p* = 0.067).

*Reliability:* The reliability of the running gait characteristics within a session was assessed using Spearman’s correlations to compare the characteristics between the first and second half of the run, as well as between the first and last minute.

For the temporal characteristics, GCT showed very strong positive correlations of rho = 0.915 (*p* < 0.05) between the halves and rho = 0.927 (*p* < 0.05) between minutes. The swing time was the least consistent between the first and second halves (rho = 0.806, *p* < 0.05) and the first and last minutes (rho = 0.782, *p* = 0.0075), demonstrating very strong, and strong positive correlations, respectively.

For the asymmetry characteristics, the correlations were generally weaker. The GC asymmetry exhibited very weak (rho = 0.079, *p* > 0.05) and moderate correlations (rho = 0.431, *p* > 0.05) between the halves and between the first and last minutes, respectively, Table 2.

### 3.2. Performance Validation

Table 3 demonstrates that there was a statistically significant difference in the performance time (*p* = 0.007) between the male and female runners at the 0.05 significance level, with the males having faster times on average. However, no significant differences were found in Age-PT (*p* = 0.108), GC (*p* = 0.130), GCT (*p* = 0.101), SWT (*p* = 0.573), or DF (*p* = 0.073).

When comparing Age-PT and the temporal characteristics, significant differences were observed for GCT (U = 0, *p* = 0.009) and DF (U = 0, *p* = 0.009) between those who had an Age-PT of above 70% (n = 4) and those who had an Age-PT under 70% (n = 6). Lower ground contact times and duty factor scores were observed in those who had Age-PT of above 70%. In contrast, the GC (U = 5, *p* = 0.171) and SWT (U = 4, *p* = 0.109) yielded no statistically significant differences between the Age-PT groups.

A Kruskal–Wallis H test was conducted to assess the differences between the groups (HS, MS, and FS) across the temporal characteristics. The results indicated no statistically significant differences between the groups. Specifically, GC showed that *H*(2) = 2.454, *p* = 0.293 and, for GCT, the results were *H*(2) = 2.510, *p* = 0.285. Similarly, SWT and DF showed that *H*(2) = 0.941, *p* = 0.625, *H*(2) = 2.510, *p* = 0.285, respectively.

## 4. Discussion

This pilot study establishes the utility of two foot-mounted IMUs for capturing and analyzing data, enabling the examination of temporal characteristics and foot strike pattern during a 5 km parkrun in ‘real-world’ environments. Furthermore, this study presents an exploratory analysis to (1) provide further analytical validation of running gait characteristics and (2) develop performance validation of running gait characteristics through differentiating between known groups. The concurrent validity of the spatiotemporal and kinematic characteristics recorded by these sensors in comparison to a 3D motion capture system have previously been reported during treadmill running at various speeds [22]. Extending their application to outdoor, uncontrolled environments with promising outcomes highlights their potential for integration into affordable commercial technologies, reducing the dependence on expert analysis and expensive gold-standard systems [36]. The methodology examined here confirms previous findings regarding extracting running gait characteristics, suitable for application in everyday or resource-limited environments. Through outdoor validation, the utilization of IMU-based techniques could contribute to comprehensive running gait analysis, furnishing relatively sparse yet extensive long-term observations.

### 4.1. Analytical Validation

The findings demonstrate face validity by highlighting observable changes in temporal characteristics throughout the run. Specifically, the increase in GCT (6.66%) and GC (2.19%) between the first and last minute aligns with expectations that runners exhibit slower gait and increased GCT as fatigue sets in [40,41,42,43]. Additionally, DF exhibited moderate increases (4.11%) between the first and second halves of the 5 km run, further suggesting shifts in the distribution of the running cycle due to fatigue. Although SWT showed minimal change, the stability of this characteristic is in line with expectations of its lesser role in fatigue-related adaptations [40,42]. The increases in asymmetry, particularly in GCT, though moderate, support the notion that fatigue impacts the symmetry of movement, albeit to a lesser extent than other temporal characteristics [44].

The analysis of the temporal characteristics in this study reveals trends consistent with findings from other research, highlighting the effects of fatigue on running mechanics [41,42,43,44,45,46]. Specifically, the slight increase in the GC time observed in this study suggests a minor slowing down as the run progresses, similar to the implications of a decreased running speed. For example, GCT showed a significant increase (+5.51% from the first to the second half and +6.66% from the first to the last minute), aligning with Meyer et al.’s findings of a +6.5% increase between 5–10 km and 25–30 km [33]. Low and consistent GCT asymmetries have been established in previous research, indicating that elite runners maintain consistent GCT despite fatigue [35]. The minor decrease in SWT in this study (−0.34% from the first to the second half and −0.35% from the first to the last minute) parallels Meyer et al.’s observed decrease of −2.2%, reflecting less time spent in the air as fatigue sets in [33]. The DF in this study showed a moderate increase (+4.11% from the first to the second half and +1.59% from the first to the last minute), which is consistent with Meyer et al.’s significant increase of +6.7%, indicating more time spent on the ground relative to the gait cycle [33].

The observed adaptations in gait characteristics across the 5 km course align partially with previous research on runners’ biomechanical responses to elevation changes (Figure 1). While the results indicated limited relationships between elevation and GCT, DF, and SWT, some significant patterns emerged, warranting further interpretation. For instance, GCT showed no significant variation with elevation changes (R^2^ = 0.000, *p* = 0.670), contrasting with findings suggesting runners increase GCT on inclines for additional propulsive force [47]. Longer GCT may facilitate greater force production per stride, which is essential for overcoming gravitational demands during ascent [48]. Similarly, DF remained unaffected by elevation (R^2^ = 0.000, *p* = 0.774), and the ANOVA results revealed no significant differences in DF across elevation bands (*p* = 0.319). These findings challenge the expectation that increased DF during uphill running reflects stability adaptations [49], possibly due to the limited effect size of elevation on these metrics. Conversely, SWT exhibited a weak but statistically significant relationship with elevation (R^2^ = 0.005, *p* = 0.032), though differences across elevation bands were not significant (*p* = 0.064). This subtle trend aligns with the notion that runners may slightly adjust the airborne time to prioritize ground contact during inclines [50]. Additionally, ASY demonstrated a weak positive relationship with elevation (R^2^ = 0.006, *p* = 0.024), potentially reflecting small compensatory adjustments in the stride symmetry under changing terrain. However, the lack of significant differences across the elevation bands (*p* = 0.361) suggests these changes are minimal. While GC exhibited a statistically significant relationship with elevation (R^2^ = 0.006, *p* = 0.012), with differences across elevation bands (*p* = 0.042), the effect size remained modest. This indicates that runners may adapt their stride duration slightly during elevation changes but not to a degree that profoundly alters the overall biomechanics. Together, these results emphasize the dynamic yet subtle nature of gait adaptations to environmental demands [39].

The degree of asymmetry in GCT between the two halves was calculated as 8.041% for the first half and 8.462% for the second half. The results obtained are notably higher compared to those from previous studies that have reported asymmetry levels of 2.6% [35], 3.5% [51], and 3.6% [52] in GCT, respectively. However, direct comparisons between these studies are challenging due to differences in methodologies (i.e., terrain, gradient, and technologies used) and the characteristics of the participants recruited. The real-world nature of the 5 km outdoor parkrun introduced variability in terrain and elevation, potentially amplifying asymmetry. By acknowledging these distinctions, we provide critical context for interpreting the results.

The study also established concurrent validity by examining correlations between the running gait characteristics and both PT and Age-PT. GCT emerged as a strong predictor of performance, showing very strong positive correlations with PT (rho = 0.918, *p* < 0.05) and negative correlations with Age-PT (rho = −0.964, *p* < 0.05). This consistency across both performance measures suggests a universal principle in running efficiency: faster runners tend to have shorter gait cycles and spend less time in contact with the ground, which contributes to a higher running economy and speed [53]. The mean DF consistently showed a strong positive correlation with PT (rho = 0.839) and a strong negative correlation with Age-PT (rho = −0.915), underscoring the significance of spending less time in ground contact for enhanced running performance [33]. In contrast, SWT and asymmetry measures, including foot strike patterns, exhibited weaker and non-significant correlations with PT and Age-PT, suggesting that these characteristics may be less influential in determining the overall performance outcomes (Table 2). The weak and non-significant correlations of the foot strike patterns with PT and Age-PT suggest that the foot strike type may not have a strong influence on the running performance in this study; the observed relationships could be due to some other mechanism (e.g., individual biomechanics, training background, or physiological adaptations) [54].

Additionally, the study assessed the reliability of these gait characteristics within a session, confirming strong internal consistency for the primary temporal characteristics. GCT exhibited the highest reliability, with very strong correlations between both the first and second halves of the run (rho = 0.915, *p* < 0.05) and between the first and last minutes (rho = 0.927, *p* < 0.05). This consistency underscores GCT’s robustness as a characteristic for analyzing the running performance across time. SWT, while somewhat less consistent, still demonstrated strong positive correlations, indicating reliable characteristic despite its minimal change throughout the run.

The asymmetry characteristics, particularly GC asymmetry, exhibited weaker reliability, with only moderate correlations between the first and last minute (rho = 0.431, *p* > 0.05), suggesting that asymmetry characteristics may be more variable and sensitive to external factors during a run (Table 2). Increases in asymmetry may indicate that the runner is adjusting their gait, possibly due to fatigue or the elevation profile of the field [55] (Table 2). The findings contrast those from previous research, in which an overall gait asymmetry is observed, but no changes in gait asymmetry over the duration of the run [35,46]. These findings collectively suggest that fatigue induces specific biomechanical adjustments during running, as reflected in the temporal characteristics, suggesting growing imbalances. These imbalances are critical as they indicate how fatigue affects coordination and stability, potentially leading to inefficient running mechanics and a higher risk of injury [40].

### 4.2. Performance Validation

The performance validation results of the gait characteristics across the groups provide insight into whether these temporal characteristics can reliably identify, quantify, or predict a meaningful physical or functional state within the specified context of use. Although the male runners demonstrated significantly faster performance times than the females (*p* = 0.007), there were no significant sex differences in the temporal gait characteristics, such as GC, GCT, DF, or SWT. This suggests that while these gait characteristics are reliable and consistent across the known groups (e.g., sex), they may not directly predict or explain performance differences based solely on temporal characteristics. For performance validation, this indicates that gait characteristics like GCT and DF are stable enough to be measured reliably across a diverse sample but might require additional contextual factors to serve as useful indicators of performance or physical states.

The significant differences observed in GCT and DF between the runners with higher (Age-PT > 70%) and lower age-graded performance scores (*p* = 0.009) are more promising from a clinical validation perspective. Specifically, the runners with higher Age-PT scores displayed shorter GCT and lower DF values, which align with patterns associated with efficient running biomechanics and potentially better musculoskeletal health and endurance [56,57]. In a clinical context, lower GCT and DF could indicate a more optimized gait pattern that minimizes joint loading and energy expenditure, which may serve as biomarkers for injury prevention, rehabilitation progress, or functional fitness assessments in active populations. Validating these characteristics as predictors of efficiency or health or performance outcomes would require further investigation, focusing on their sensitivity to detect changes in gait due to intervention or the progression of a clinical condition [18].

At present, there is inconclusive evidence in support of the non-rearfoot strike (RFS) pattern (here, MS or FS) conferring a competitive advantage over the RFS pattern (HS) pattern [58,59]. The results observed here support this, as no statistically significant differences were found between the temporal characteristics and foot strike pattern. Thus, this suggests that the foot strike pattern may not have a substantial influence on these specific temporal characteristics and subsequent performance outcomes within this sample, or that other factors might play a more dominant role in the performance outcomes. This insight underscores the importance of selecting gait characteristics that are sensitive to the specific research (biomechanical) question. For instance, if foot strike is associated with certain injury profiles or adaptations, it may require different or additional characteristics beyond temporal characteristics to fully capture its impact on gait health. For example, FS and MS often experience increased loading on the Achilles tendon and calf musculature, which can lead to a different injury profile, including Achilles tendinopathy and metatarsal stress fractures [60]. These adaptations may not be effectively captured by temporal characteristics (e.g., GCT or DF) alone. Instead, additional biomechanical characteristics—such as the vertical loading rate, knee flexion angle at initial contact, and ankle plantarflexion angle—are necessary to fully understand the relationship between the foot strike pattern and injury risk [54].

### 4.3. Limitations

Firstly, the study sample size was relatively small, consisting of only 10 participants. This limited sample size may restrict the generalizability of the findings and the ability to detect smaller or more nuanced effects. Additionally, individual asymmetry is obscured when data are averaged across a sample, as demonstrated in the current study. Therefore, in performance settings, it is essential to maintain personalized data assessment when assessing an athlete’s gait for diagnostic and prognostic purposes.

Moreover, although the 5 km run was completed on a standardized course, the terrain and gradient varied throughout the run, thus limiting the ability to comprehensively assess the impact of fatigue on temporal characteristics. Gradient changes can significantly affect running biomechanics, including the stride length, cadence, and foot strike patterns [55].

The age grade performance offers a standardized approach to compare performances across age and gender, yet it may overlook individual differences in training and fitness levels, as well as external factors like weather and terrain. Moreover, it assumes an “ideal” runner for each age and sex category, which might not accurately represent the capabilities of all runners. The accuracy of age grade performance calculations depends on the quality and availability of the race data, leading to varying levels of precision. While correlations between PT and Age-PT remain consistent in their direction, their strength varies slightly across different characteristics (GC, GCT, and SWT). PT directly reflects the 5 km run time, potentially influenced by gait characteristics’ impact on speed, while the age grade scores adjust for age and sex, facilitating demographic comparisons. This adjustment may either enhance or diminish the direct influence of gait characteristics, contingent upon the dataset’s age and sex distribution.

## 5. Conclusions and Future Work

This study highlights the utility of foot-mounted IMUs for conducting a reliable and valid running gait analysis in real-world settings. The findings underscore the robustness of temporal characteristics like the ground contact time and duty factor in detecting fatigue and assessing running performance, with both characteristics showing clear correlations with higher efficiency in runners. Despite some limitations in the reliability of gait asymmetry, the overall performance validation confirms the capability of these IMU-based measurements to distinguish key aspects of running biomechanics. Furthermore, the observed consistency in the temporal characteristics across genders, combined with significant differences in the age-graded performance, suggests that these characteristics can serve as universal indicators of the running efficiency, independent of sex. These findings affirm the value of IMU-based technology as an accessible and effective tool for monitoring running performance and fatigue, with broad potential for applications in both sports medicine and athletic training.

Future research should aim to expand the validity profiling of the Ax6 sensors, by incorporating larger and more diverse samples. This would provide a more comprehensive understanding of the relationship between temporal characteristics, foot strike types, age grade running performance across different demographics, skill levels, injury histories, and training backgrounds. Integrating GPS data with IMU outputs could further enhance insights into running biomechanics across varied terrains and gradients. Although GPS has limited spatial accuracy, its ability to approximate terrain and gradient is critical for interpreting gait changes and fatigue dynamics in outdoor settings.

Longitudinal studies could also play a pivotal role in leveraging the capabilities of IMU technology by tracking long-term gait data to identify asymmetries and strength deficits linked to injury risk [61]. For example, while asymmetry levels of up to 4% are considered normal [62], critical levels exceeding 15% could signal a heightened injury risk [63]. Such data could also support monitoring rehabilitation progress and inform personalized interventions in sports and clinical contexts.

## Figures and Tables

**Table 1:** Timed characteristics compared to mean temporal gait characteristics and calculated age grade performance for each runner.

ID	Sex	Age	PT (min)	Age-PT (%)	GC (s)	GCT (s)	SWT (s)	DF	HS (%)	MS (%)	FS (%)	Predominant Foot Strike Pattern
1	F	26	20.19	72.52	0.677	0.158	0.519	23.118	33.743	51.958	14.297	MS
2	M	35	16.45	77.96	0.605	0.118	0.486	19.422	32.242	19.333	48.424	FS
3	M	28	20.19	63.25	0.685	0.185	0.500	26.930	25.329	51.860	22.810	MS
4	F	38	27.27	54.60	0.654	0.222	0.428	34.202	26.471	43.290	30.238	MS
5	M	30	17.15	74.49	0.657	0.111	0.546	16.748	31.226	41.200	27.573	MS
6	F	27	23.01	64.01	0.679	0.169	0.510	24.656	30.169	51.494	18.335	MS
7	F	32	26.26	55.75	0.723	0.187	0.536	25.749	36.221	45.352	18.425	MS
8	F	26	20.27	72.05	0.679	0.149	0.520	21.397	37.692	32.572	29.734	HS
9	F	57	33.07	53.88	0.750	0.249	0.496	34.081	28.695	47.712	23.591	MS
10	F	53	32.26	52.55	0.746	0.253	0.491	34.576	22.950	45.861	31.187	MS
Average		35.2	23.43	62.49	0.685	0.180	0.503	26.692	31.745	42.354	25.899	MS

**Table 2:** The percentage change in the mean and asymmetry of temporal characteristics during the initial and latter halves and first and last minutes of the 5 km run. Spearman’s (rho) correlation coefficients for the relationship between performance time (PT) and age grade performance time (Age-PT) with various gait characteristics, including temporal parameters, gait asymmetry, and foot strike patterns. * denotes statistically significant correlations (*p* < 0.05).

	Temporal Characteristics	First Half	Second Half	Change (%)	rho	First Minute	Last Minute	Change (%)	rho	PT rho	Age-PT rho
**Mean**	Gait cycle*(s)*	0.684	0.691	1.01	0.878 *	0.673	0.688	2.19	0.756 *	0.681 *	−0.745 *
	Ground contact time*(s)*	0.174	0.185	5.51	0.915 *	0.165	0.177	6.66	0.927 *	0.918 *	−0.964 *
	Swing time*(s)*	0.508	0.506	−0.34	0.806 *	0.513	0.511	−0.35	0.782	−0.14	0.164
	Duty factor	25.291	26.376	4.11	0.903 *	24.074	24.464	1.59	0.854 *	0.839 *	−0.915 *
**Asymmetry**	Gait cycle*(s)*	1.489	1.496	0.46	0.079	1.587	1.549	−2.44	0.431	−0.067	0.224
	Ground contact time*(s)*	8.041	8.462	5.24	0.244	8.064	8.281	2.70	0.576	−0.122	0.176
	Swing time*(s)*	2.291	2.381	3.91	0.218	2.371	2.477	4.48	0.523	0.061	0.018
	Duty factor	7.178	7.232	0.75	0.302	6.971	7.209	3.42	0.498	0.094	0.034

**Table 3:** Mann–Whitney U test comparing male vs. female runners *.

	Characteristic	Female	Male	U	*p*
Mean	Gait cycle(s)	0.698	0.651	16	0.266
	Ground contact time(s)	0.194	0.132	17	0.183
	Swing time(s)	0.504	0.513	9	0.833
	Duty factor	27.671	20.658	17	0.183
Asymmetry	Gait cycle (s)	3.673	7.761	9	0.833
	Ground contact time (s)	7.682	9.012	9	0.833
	Swing time (s)	6.824	7.943	9	0.833

* Correlation is significant at the 0.05 level (2-tailed).

## Data Availability

Data will be available from the study upon reasonable request from the corresponding author.
